# The Wisconsin Decision

**Published:** 1899-12

**Authors:** 


ARTICLE VI.

### THE WISCONSIN DECISION.

Those who have carefully read our last issue must have been convinced that the policy of this magazine is to view all dental questions without favor or favoritism. In the past, at times, because we have expressed the view that the Examiners rather than the Faculties, were in the right, some have thought that Items of Interest was an "organ" of the Examiners Association. Nothing could be farther from the truth. In our July number we gave place to an article accusing the Illinois Board of Examiners of irregular practices. This does not mean, however, that we assume all the charges to be true. Our publication of the matter is rather in the nature of the finding of an indictment by a Grand Jury, an act which does not assert that there is guilt, but that there is evidence against the accused which in his own interest must be refuted before he can be counted innocent. The rumors from Illinois are of such a nature that the good name of dentistry demands an investigation, and we trust that after the Niagara meetings they will be hushed finally.

We come now to a discussion of the so-called Wisconsin decision, in a dispassionate, impartial manner, in the hope of determining how much or how little it affects the dental laws of the whole country. In certain quarters the decision has been welcomed and the conclusion advanced that the Judge's opinion negatively settles the question whether or not an Examining Board may determine as to the status and reputability of the colleges. But does it?

It seems safe to assert that it does not. All that was before the Court for consideration was the powers of the *Wisconsin* Board, as defined by a *Wisconsin* statue, and a close study of the decision shows that all that is decided (or claimed to be decided) is that the powers of this particular board are not as great as the Board claimed. This opinion is not even final in Wisconsin, the case as yet not having been tried. The Judge's opinion, however, seems so sound that there is little reason to believe that it would be overturned by a higher court, although it is evident that he must have had a very foggy notion of certain phases of the questions at issue, for he tells us that the Board in effect demanded that to be counted reputable, colleges must teach Latin and Mathematics, and a *reductio ad absurdum* argument declares that if this power were granted the Board might in the future demand a course in Chinese or Hindoo.

In considering the bearing of this decision upon boards in other states we must study the statue, and before it could be claimed that the opinion could serve as a legal precedent, it would be needful to show a likeness between this particular statute and those of other states. It will be instructive to do this, and for comparison we select the law of California; first, because of its seeming similarity; and, second, because of the quite opposite decision of the California Supreme Court, a decision which it seems to have been convenient for some to forget.

We give the two laws, and italicize the lines which make them so different that opposite judicial decisions have resulted.

"Any person who may desire such a license, may appear before the State Board of Dental Examiners at any regular meeting and be examined with reference to his knowledge and skill in dental surgery; if such examination shall be satisfactory the Board shall issue a license to practice dentistry; provided that the Board *shall license without examination*, upon the payment of one dollar, *any regular graduate of an incorporated and reputable dental college* which requires that the candidate for graduation shall attend two full courses of lectures of five months each, the last of which courses shall be attended in the college which issues the diploma."

"Any and all persons who shall so desire may appear before said Board at any of its regular meetings, and be examined with reference to their knowledge and skill in dental surgery, and if the examinations of any such person or persons shall prove satisfactory to said Board, the Board of Examiners shall issue to such persons as they shall find to possess the requisite qualifications, a certificate to that effect in accordance with the provisions of this act. Said Board shall also indorse as satisfactory diplomas from any reputable dental college, *when satisfied of the character of such institution* upon the holder furnishing evidence *satisfactory to the Board* of his or her right to the same, and shall issue certificates to that effect within ten days after."

In the Wisconsin case the Board claimed that the legislature had clothed them with power to decide as to the reputability of the colleges. The whole question turned on this single point, and the Judge found that the legislature gave the Board no such power. Nor does it seem to require much legal acumen to arrive at the same view. The language of the statute seems simple. It directs the Board to license graduates of reputable schools. This is *mandatory*. Should the Board reject a graduate, claiming that his school lacked reputability, and should the rejected candidate take issue with the Board, the reputability of the school would become a question of fact upon which the court could rightfully hear evidence; this because there is nothing in the statute of *Wisconsin* which authorizes the Board to decide what is or is not reputable. The whole argument of the Judge in his opinion is upon this point, and it was because, in his view, the reputability of the Chicago College was a long established and recognized fact that he decided against the Board. It is worthy of note that the statute does give the Board certain supervisory powers. The Judge declares that the Board could not direct the College to demand certain educational standards of admission, and he is right because the statute is silent on this point. But he would certainly have decided that the Board could demand that the college should have "two full courses of five months each," because the statute specifically empowers the Board to do this.

Thus we find that the Wisconsin decision does not establish a principle of equity, but merely a point of statute law. The Board claimed certain discretionary powers, and the Judge decides that the statute does not bequeath such powers. That is absolutely all that the Wisconsin decision decides, and the effect of the decision is limited to Wisconsin, and can only effect such states as have the same language in their statutes.

The language of the California law is very similar, but in regard to the reputability of colleges we find here the significant phrase, "*when satisfied of the character of such institution.*" Thus the California Board is made the final arbiter of the reputability of colleges sending candidates to it for license, and this power being granted by statute, the Supreme Court has decided that the court has no supervision over the Board on this point. Throughout his opinion the California Judge repeatedly quotes the statute to maintain his view that everything must be *satisfactory* to the Board. Had the farmers of the Wisconsin law inserted such a saving clause there is no doubt that the same Judge who decided against them would have decided in the favor.

Apparently (the decision of the Supreme Court of California sustaining this view), a state may enact police regulations defining who shall or who shall not practice a profession within its borders, and as a part of such requirement it may demand not alone certain educational attainments determinable by examination, but may go further and relieve its examiners of some of their labor by extracting that licentiates must come from colleges requiring specified knowledge as a preliminary to matriculation. Where such provision does not appear in the statute, of course the Board cannot exercise any such powers. As the Wisconsin Board appear to have exceeded their law they were "thrown out of court."—*Editorial in Items of Interest.*

